# Cancer history, bandemia, and serum creatinine are independent mortality predictors in patients with infection-precipitated hyperglycemic crises

**DOI:** 10.1186/1472-6823-13-23

**Published:** 2013-07-16

**Authors:** Chien-Cheng Huang, Willy Chou, Hung-Jung Lin, Shih-Chung Chen, Shu-Chun Kuo, Wei-Lung Chen, Jiann-Hwa Chen, Hsien-Yi Wang, How-Ran Guo

**Affiliations:** 1 Department of Emergency Medicine, Chi-Mei Medical Center, Tainan, Taiwan; 2 Department of Child Care and Education, Southern Taiwan University of Science and Technology, Tainan, Taiwan; 3 Department of Environmental and Occupational Health, Medical College, National Cheng Kung University, Tainan, Taiwan; 4 Department of Physical Medicine and Rehabilitation, Chi-Mei Medical Center, Tainan, Taiwan; 5 Department of Recreation and Health Care Management, Cha Nan University of Pharmacy and Science, Tainan, Taiwan; 6 Department of Biotechnology, Southern Taiwan University, Tainan, Taiwan; 7 Department of Electrical Engineering, Southern Taiwan University of Science and Technology, Tainan, Taiwan; 8 Department of Ophthalmology, Chi-Mei Medical Center, Tainan, Taiwan; 9 Department of Optometry, Chung Hwa University of Medical Technology, Tainan, Taiwan; 10 Department of Emergency Medicine, Cathay General Hospital, Taipei, Taiwan; 11 Fu Jen Catholic University School of Medicine, Taipei, Taiwan; 12 Department of Nephrology, Chi-Mei Medical Center, Tainan, Taiwan; 13 Department of Sport Management, College of Leisure and Recreation Management, Chia Nan University of Pharmacy and Science, Tainan, Taiwan

**Keywords:** Hyperglycemic crises, Hyperosmolality, Infection, Mortality, Predictor

## Abstract

**Background:**

Infection is the most common precipitating factor and cause of death in patients with hyperglycemic crises. Treating infection-precipitated hyperglycemic crises includes using empiric antibiotics early; correcting dehydration, hyperglycemia, and electrolyte imbalances; and frequent monitoring. Intensive care unit admission, broad-spectrum antibiotics, and even novel therapy for infection may be beneficial for patients with a high risk of mortality. However, these management options are costly and not beneficial for every patient. Selecting high-risk patients who would most likely benefit is more appropriate. We investigated the independent mortality predictors of patients with infection-precipitated hyperglycemic crises to facilitate clinical decision making.

**Methods:**

This study was conducted in a university-affiliated medical center. Consecutive adult patients (> 18 years old) visiting the Emergency Department between January 2004 and December 2010 were enrolled when they met the criteria of an infection-precipitated hyperglycemic crisis. Thirty-day mortality was the primary endpoint.

**Results:**

One hundred forty-two patients were enrolled. The infection source did not predict mortality. The presenting variables that were independently associated with 30-day mortality in a multiple logistic regression model were cancer history (odds ratio [OR], 7.4; 95% confidence interval [CI], 2.4-23.2), bandemia (OR, 7.0; 95% CI, 1.6-30.3), and serum creatinine (OR, 1.4; 95% CI, 1.1-1.8). The common sources of infection were the lower respiratory tract (30.3%), urinary tract (49.3%), skin or soft tissue (12.0%), and intra-abdominal (6.3%).

**Conclusions:**

Cancer history, bandemia, and serum creatinine level are three independent mortality predictors for patients with infection-precipitated hyperglycemic crises. These predictors are both readily available and valuable for physicians making decisions about risk stratification, treatment, and disposition.

## Background

Hyperglycemic crises present a disease continuum of diabetic emergency. The basic underlying mechanism is the combination of absolute or relative insulin deficiency and an increase in counter regulatory hormones: glucagon, catecholamines, cortisol, and growth hormone [1]. There are three types of hyperglycemic crisis: (a) diabetic ketoacidosis (DKA), (b) hyperosmolar hyperglycemic state (HHS) (a and b are two extremes of the same clinical syndrome), and (c) mixed syndrome (both DKA and HHS as a mixed state of acidosis and hyperosmolality) [2-7].

Despite recent improvements, the incidence and the cost of treating hyperglycemic crises are high and continue to rise. The annual DKA incidence rate has been estimated in population-based studies to range from 4.6 to 8 episodes per 1,000 patients with diabetes, and recent epidemiological studies in the U.S. report that the annual DKA incidence rate sharply increased during the past two decades [3]. In 2006, there were about 136,510 hospitalizations for DKA in the U.S. [8]. The average cost per patient per hospitalization was US$13,000, and the annual medical expenditure for healthcare providers to patients with DKA might exceed US$1 billion [3]. The incidence of and medical expenditure for HHS care are unknown because there are few population-based studies on HHS, and because many patients with HHS have multiple comorbidities. The rate of hospital admissions for HHS was estimated more than a decade ago to be 1% of all primary diabetic admissions [9]. The mortality rate for hyperglycemic crises remains high: 1-9% for DKA, 5-45% for HHS, and 5-25% for mixed DKA/HHS [1,4,5]. Among the elderly (≥ 65 years old), the mortality rate was recently reported to be as high as 71% [10].

Infection is the most common precipitating factor in patients with hyperglycemic crises. Estimates range from 32% to 60%, with urinary tract infection, pneumonia, and sepsis as the most frequent types [11]. Other precipitating factors are inadequate or inappropriate therapy, pancreatitis, myocardial infarction, cerebrovascular accident, and drugs [1]. Infection is also the most common (67-80%) cause of death [5,12,13]. Therefore, investigating and treating infection is the first priority when a patient presents with a hyperglycemic crisis. Thresholds for blood cultures and empiric antibiotics should be low because the manifestation of infection may be unapparent, especially in long-term diabetes and in elderly patients [1,14].

Treating infection-precipitated hyperglycemic crises includes using empiric antibiotics early; correcting dehydration, hyperglycemia, and electrolyte imbalances; and frequent monitoring [1]. Intensive care unit admission, broad-spectrum antibiotics, and even novel therapy for infection may be beneficial for patients with a high risk of mortality [15]. However, these management options are costly and not beneficial for every patient. Selecting high-risk patients who would most likely benefit is more appropriate. There is no published study about this, however. Therefore, our goals were to (i) identify univariate correlates of death in patients with infection-precipitated hyperglycemic crises, and (ii) identify independent mortality predictors that may help clinical decision making.

## Methods

### Study design, setting, population, and selection of participants

This study was done in a 700-bed university-affiliated medical center in Taipei with a 40-bed emergency department (ED) staffed with board-certified emergency physicians who provide emergency care to approximately 55,000 patients per year. Consecutive adult patients (> 18 years old) visiting the ED between January 2004 and December 2010 were enrolled when they met the following criteria [11]: (i) DKA was defined as casual plasma glucose > 250 mg/dL, a high anion gap metabolic acidosis (anion gap > 10, serum HCO_3_ < 18 mmol/L, and pH < 7.3), and positive urine ketones or serum ketones; (ii) HHS was defined as casual plasma glucose > 600 mg/dL, increased effective serum osmolality > 320 mOsm/kg, anion gap < 12, no significant acidosis (HCO_3_ > 15 mmol/L or pH > 7.3), small urine ketones or serum ketones, and alteration in mental state; (iii) Mixed syndrome (DKA plus HHS) was defined as acidosis (pH < 7.3, HCO_3_ < 18 mmol/L), positive urine ketones or serum ketones, and effective serum osmolality > 320 mosm/kg. The effective serum osmolality was calculated using the formula:

$$2\left[\mathrm{measured}\;Na^{+}\left(\mathrm{mEq}/L\right)\right]+\left[\mathrm{glucose}\left(\mathrm{mg}/\mathrm{dL}\right)\right]/18\;\left[11\right]$$

The definition of infection included lower respiratory tract infection, urinary tract infection, intra-abdominal infection, skin or soft tissue infection, meningitis, bone/joint infection, perianal abscess, psoas muscle abscess, and sepsis without focus. The clinical impression of infection was based on the diagnoses of the treating physician’s documentation, laboratory, and image results (such as pneumonia on a chest radiograph, pyuria on a urinary analysis, abscess on computed tomography, etc.).

### Data collection and definition of variables

All treatment of hyperglycemic crises in the studied hospital is strictly according to the guidelines suggested by the American Diabetes Association [1,3,11]. Patients were prospectively selected in the ED. Insufficient information was retrospectively collected by reviewers checking medical records after the patients had been discharged from the hospital. The studied hospital’s Human Investigation Committee approved the protocol. The reviewers were blinded to the patients’ hospital courses and outcomes. Information for a number of variables for each patient was recorded (Tables 1 and 2). Cancer history was defined as any mention of metastatic or non-metastatic cancer in a patient’s medical records. Bandemia was defined as greater than 10% band forms [16]. Any variable not noted in history or physical exam as present was considered to be absent.

**Table 1:** Univariate analysis of clinical variables of 142 patient visits with hyperglycemic crises precipitated by infection

**Variable**	**Survival (n = 115)**	**30-day mortality (n = 27)**	***P*-value**	**All (n = 142)**
Age, mean ± SD	67.5 ± 17.1	72.4 ± 19.7	0.200	68.4 ± 17.6
Elderly (≥ 65 years old), %	62.6	81.5	0.073	66.2
Gender: Male, %	37.4	48.1	0.303	39.4
Altered mental status, %	53.0	77.8	0.029	57.7
SBP, mean ± SD	135.0 ± 32.0	127.5 ± 36.7	0.289	133.6 ± 32.9
Heart rate, mean ± SD	118.1 ± 21.3	106.0 ± 26.3	0.013	115.8 ± 22.8
Body temperature, mean ± SD	37.3 ± 1.2	37.1 ± 1.4	0.387	37.2 ± 1.2
Respiratory rate, mean ± SD	22.1 ± 6.1	21.9 ± 6.5	0.898	22.1 ± 6.1
Medical history, %				
Hypertension	53.9	48.1	0.589	52.8
Diabetes	82.6	88.9	0.567	83.8
Stroke	33.0	33.3	>0.95	33.1
Chronic renal insufficiency	14.8	25.9	0.166	16.9
Cancer	7.8	29.6	0.005	12.0
Bedridden	20.0	25.9	0.600	21.1
Infection source, %^*^				
Low respiratory tract	31.3	25.9	0.649	30.3
Urinary tract	52.2	37.0	0.157	49.3
Skin or soft tissue	10.4	18.5	0.319	12.0
Intra-abdominal	5.2	11.1	0.372	6.3
Meningitis	0.9	0.0	>0.95	0.7
Bone/joint	0.9	0.0	>0.95	0.7
Perianal abscess	0.0	3.7	0.190	0.7
Psoas muscle abscess	0.9	0.0	>0.95	0.7
Sepsis without focus	0.9	0.0	>0.95	0.7
Subgroup diagnosis, %				
DKA	22.6	14.8	0.443	21.1
HHS	64.3	66.7	>0.95	64.8
Mixed DKA/HHS	13.0	18.5	0.538	14.1

*SD* standard deviation, *SBP* systolic blood pressure, *DKA* diabetic ketoacidosis, *HHS* hyperosmolar hyperglycemic state.

^*^Patient may have multiple infection sources.

**Table 2:** Univariate analysis of biochemical variables of 142 patient visits with hyperglycemic crises precipitated by infection

**Variable**	**Survival (n = 115)**	**30**-**day mortality (n = 27)**	***P*-value**	**All (n = 142)**
Laboratory data, mean ± SD				
Blood glucose (mg/dL)	739.9 ± 311.0	757.3 ± 259.8	0.788	743.2 ± 301.1
WBC (cells/mm^3^)	15300.0 ± 6406	13700.0 ± 5909	0.250	15000.0 ± 6324
Hemoglobin (g/dL)	13.1 ± 2.6	12.5 ± 3.0	0.314	12.9 ± 2.7
Platelet (1000/mm^3^)	247.5 ± 103.1	203.5 ± 82.2	0.041	239.2 ± 100.7
Osmolarity (mOsm/kg)^*^	335.6 ± 33.0	335.4 ± 28.6	>0.95	346.5 ± 34.5
Serum creatinine (mg/dL)	2.2 ± 1.4	3.3 ± 2.7	0.046	2.4 ± 1.8
Blood pH^†^	7.4 ± 0.1	7.3 ± 0.2	0.208	7.4 ± 0.1
HbA1c (%)^‡^	11.2 ± 3.2	10.1 ± 2.7	0.183	11.0 ± 3.1
Bandemia (> 10% band), %	4.3	14.8	0.047	6.3

*SD* standard deviation, *WBC* white blood cell count.

^*^Effective serum osmolarity: 2[measured Na^+^ (mEq/L)] + [glucose (mg/dL)]/18.

^†^90.1% (128/142) patients had this test.

^‡^72.2% (83/115) survival patients had this test; 66.7% (18/27) 30-day mortality patients had this test.

The enrolled patients were divided into two groups based on their 30-day outcome: (i) 30-day mortality and (ii) survival. All the study variables were used for comparisons between groups.

### Definition of endpoint

We used the 30-day mortality as the primary endpoint. People who survived at least 30 days whether or not they were still hospitalized were considered “survival” for this analysis.

### Data analysis

All analyses were done using SPSS 16.0 for Windows (SPSS Inc., Chicago, IL, USA). Continuous data are means ± SD. Comparisons between two groups were made using either an independent-samples *t*-test (assuming normal distribution) or Mann–Whitney/Wilcoxon tests (assuming non-normality) for the continuous variables. Either a χ^2^ test or a Fisher’s exact test was used for categorical variables. One-way ANOVA was used in comparisons between the subgroups of hyperglycemic crises. The variables in the univariate comparison (P < 0.1) were then included in a multiple logistic regression analysis of risk for 30-day mortality. Statistical significance was set at P < 0.05 (two tailed).

## Results

During our study period, 160 patients with hyperglycemic crises precipitated by infection presented to the ED. Eighteen were excluded from the study because of insufficient data or because they had been treated in another hospital.

**Figure 1:**
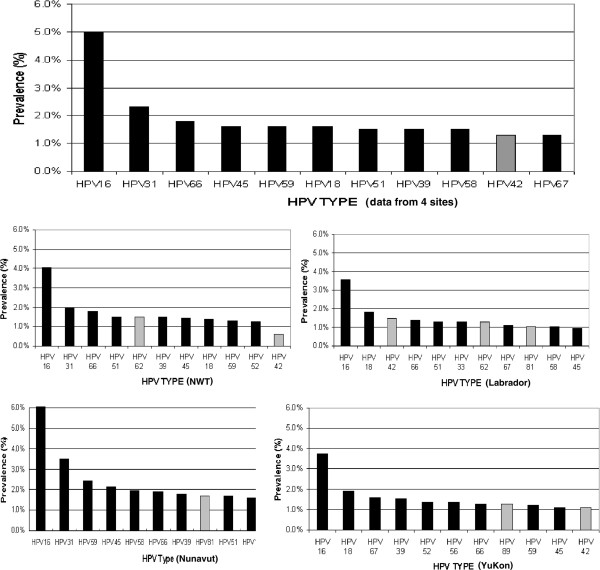
Comparison of patient number of DKA, HHS, and mixed DKA/HHS among survivors and 30-day mortality.

The final study cohort consisted of 142 patients (56 [39.4%] men, 86 women [60.6%] age range: 19 to 93 years old; mean age ± SD: 68.4 ± 17.6 years; median: 73 years) (Table 1). Twenty-seven (19%) of them died within 30 days. The percentages of DKA, HHS, and mixed DKA/HHS were 30/142 (21.1%), 92/142 (64.8%), and 20/142 (14.1%), respectively. The distribution of DKA, HHS, and mixed DKA/HHS among survivors and 30-day mortality was showed in Figure 1.

Univariate analysis was used to compare survival and 30-day mortality (Table 1). The common sources of infection were low respiratory tract (30.3%), urinary tract (49.3%), skin or soft tissue (12.0%), and intra-abdominal (6.3%). Multiple logistic regression modeling was then done using the univariate comparison P < 0.1 (Tables 1, 2, 3). The infection source did not predict mortality. The presenting variables independently associated with 30-day mortality in the multiple logistic regression model were cancer history (odds ratio [OR], 7.4; 95% confidence interval [CI], 2.4-23.2), bandemia (OR, 7.0; 95% CI, 1.6-30.3), and serum creatinine (OR, 1.4; 95% CI, 1.1-1.8).

**Table 3:** **Multivariate logistic regression modeling using a univariate comparison** (***P*** < **0**.**1**)

**Variable**	**Odds ratio (95% Confidence Interval)**		***P*-value**
	**Full model**	**Final model**	
Cancer history	4.5 (1.3-15.4)	7.4 (2.4-23.2)	0.001
Bandemia (> 10% band)	6.2 (1.1-33.1)	7.0 (1.6-30.3)	0.010
Serum creatinine	1.4 (1.1-1.7)	1.4 (1.1-1.8)	0.007
Elderly (≥ 65 years old)	2.1 (0.6-7.5)	NA	
Altered mental status	2.6 (0.8-8.2)	NA	
Heart rate	0.9 (0.8-1.0)	NA	
Platelet (1000/mm^3^)	0.9 (0.8-1.0)	NA	

*NA* not available; variable not included in the final model.

## Discussion

This study delineated independent mortality predictors of the adult patients with hyperglycemic crises precipitated by infection. Multiple logistic regression analysis showed that cancer history, bandemia, and serum creatinine level were the independent mortality predictors. These predictors, readily available to physicians, can provide valuable information for the risk stratification, treatment, and disposition of patients. For patients with more mortality predictors, aggressive intervention—intensive care unit (ICU) admission, fluid resuscitation, source identification and control, broad-spectrum antibiotics, and novel therapy for infection such activated protein C should be considered [15,17].

Metastatic cancer has been used as a mortality predictor in ICU mortality scores such as the MPM (Mortality Probability Model), SAPS (Simplified APACHE Score), and APACHE (Acute Physiology and Chronic Health Evaluation) [18]. In this study, “cancer history” included the presence of any malignancy, whether metastatic or non-metastatic. We found that this “cancer history” variable was a better predictor than was metastatic cancer. Elevated serum creatinine indicates a poor prognosis, which is more likely due to renal impairment rather than infection. Bandemia refers to an excess of band cells (immature white blood cells) released by the bone marrow into the blood. It signifies infection (or sepsis) or inflammation. A band level > 10% is a criterion of sepsis [16] and has been identified as a mortality predictor in sepsis [19]. We also found that it was an accurate predictor of mortality.

In clinical practice, blood PH and effective serum osmolality are important for evaluating hyperglycemic crises. Interestingly, these two factors were not mortality predictors (Table 2). In other words, more acidosis and higher osmolality do not correlate with a poorer outcome. In previous studies [1,4,5], the mortality rates of the three different types of hyperglycemic crisis (DKA, HHS, and mixed syndrome DKA/HHS) were different. In the present study, the overall 30-day mortality rates of DKA, HHS, and mixed syndrome DKA/HHS were 4/30 (13.3%), 18/92 (19.6%), and 5/20 (25%), respectively. We found differences in mortality similar to the literature but this did not reach statistical significance, likely due to small group sizes. The subgroup diagnosis was also not a mortality predictor.

The causes of death were further analyzed. Most of the patients died from infection (25/27; 92.6%), not the hyperglycemic crisis itself. Two patients died from ventricular arrhythmia related to hypokalemia; both were young (23 and 26 years old) women with type 1 diabetes.

This study has several limitations. First, some data were collected from a retrospective chart review. These clinical presentations or records may not have been completely documented. Second, this was a single-center study. Findings from our database may not be generalizable to other cohorts in Taiwan or in other nations. Third, the interpretation of infection in an early stage may be different between physicians. In a busy ED with a short patient stay, suspected infection by the treating physician along with laboratory and image result is more practical than confirmed infection. Fourth, there is a dramatically high prevalence of HHS relative to DKA in this population and this may be why the pH did not correlate with death, since the majority in this small cohort did not have acidosis. In our previous study of the same population [20], DKA constituted 31.5% of all hyperglycemic crises, which was compatible with the observations by Wachtel et al. in Rhode Island [2] and by Chung et al. in Jamaica [4]. Therefore, the high prevalence of HHS relative to DKA may be due to the fact that this study enrolled a subgroup of patients who had infection-precipitated hyperglycemic crises, and therefore the ratio of HHS to DKA cases was not the same as in the whole patient population. Fifth, the number of patients might have been too small to draw any firm conclusions, and further study with a much larger study population will be necessary to clarify this issue.

## Conclusions

Cancer history, bandemia, and serum creatinine level are independent mortality predictors for patients with infection-precipitated hyperglycemic crises. These predictors are both readily available and valuable for physicians making decisions about risk stratification, treatment, and disposition.

## Competing interests

This study was supported by grant CMNCKU10216 from the Chi-Mei Medical Center.

## Authors’ contributions

HCC, WHY, GHR, and CW conceived the study concept and design, acquired data, did statistical analysis, analyzed and interpreted the data, wrote the manuscript, and reviewed and edited the manuscript. LHJ, CSC, and KSC reviewed and edited the manuscript. CWL and CJW acquired, analyzed, and interpreted data. HCC takes responsibility for the paper as a whole. All authors have read and approved the final manuscript.

## Pre-publication history

The pre-publication history for this paper can be accessed here:

http://www.biomedcentral.com/1472-6823/13/23/prepub
